# Tumor suppressor miR-24 restrains gastric cancer progression by downregulating RegIV

**DOI:** 10.1186/1476-4598-13-127

**Published:** 2014-05-28

**Authors:** Yantao Duan, Lei Hu, Bing Liu, Beiqin Yu, Jianfang Li, Min Yan, Yingyan Yu, Chen Li, Liping Su, Zhenggang Zhu, Ming Xiang, Bingya Liu, Qiumeng Yang

**Affiliations:** 1 Shanghai Key Laboratory of Gastric Neoplasms, Department of Surgery, Shanghai Institute of Digestive Surgery, Ruijin Hospital, Shanghai Jiao Tong University School of Medicine, No 197 Ruijin er Road, Shanghai 200025, People’s Republic of China

**Keywords:** miR-24, RegIV, Gastric cancer, Proliferation, Invasion, Metastasis

## Abstract

**Background:**

microRNAs are small noncoding RNAs that modulate a variety of cellular processes by regulating multiple targets, which can promote or inhibit the development of malignant behaviors. Accumulating evidence suggests miR-24 plays important roles in human carcinogenesis. However, its precise biological role remains largely elusive. This study examined the role of miR-24 in gastric cancer (GC).

**Methods:**

The expression of miR-24 in GC tissues compared with matched non-tumor tissues and GC cells was detected by qRT-PCR. Synthetic short single or double stranded RNA oligonucleotides and lentiviral vectors were used to regulate miR-24 expression in GC cells to investigate its function *in vitro* and *in vivo*.

**Results:**

miR-24 was significantly downregulated in GC tissues compared with matched non-tumor tissues and was associated with tumor differentiation. Ectopic expression of miR-24 in SGC-7901 GC cells suppressed cell proliferation, migration and invasion *in vitro* as well as tumorigenicity *in vivo* by inducing cell cycle arrest in G0/G1 phase and promoting cell apoptosis. Furthermore, we identified RegIV as a target of miR-24 and demonstrated that miR-24 regulated RegIV expression via binding its 3′ untranslated region.

**Conclusions:**

miR-24 functions as a novel tumor suppressor in GC and the anti-oncogenic activity may involve its inhibition of the target gene RegIV. These findings suggest the possibility for miR-24 as a therapeutic target in GC.

## Background

Gastric cancer (GC) is one of the most common human cancers. Although the incidence and mortality have decreased worldwide over the past 20 years, GC still ranks as the fourth most common and the second most lethal cancer worldwide [1]. Although research in GC has made great progress, the molecular mechanisms underlying GC still have not been fully elucidated.

MicroRNAs (miRNAs) are small noncoding, double-stranded RNA molecules that can modulate the expression of target genes by influencing the post-transcriptional processes regulating gene expression. miRNAs recognize target mRNAs based on complete or incomplete sequence complementarity and prevent the production of protein by binding to the 3′ untranslated region (UTR) or the open reading frame (ORF) of target mRNA [2,3]. miRNAs have been demonstrated to function in cellular proliferation and differentiation processes, including the epithelial-mesenchymal transition [4] and endodermal differentiation of human embryonic stem cells [5]. In addition to general cellular functions of miRNA, these molecules also play important roles in a variety of human diseases. Multiple studies have shown that the misregulation of miRNAs is related with cancer [6,7], in which they function as either oncogenes or tumor suppressors. miRNAs have been reported to be involved in acute myeloid leukemia, breast cancer, non-small cell lung carcinoma, hepatocellular carcinoma, colon cancer, GC and other cancers [8-13]. Overexpression or downregulation of miRNAs leads to diversification of protein expression, therefore contributing to tumorigenesis by modulating proliferation, angiogenesis and invasion. Researchers found that miRNA expression profiles can be used to classify human cancers, and this finding was prior to the use of messenger RNA profiles for classification of poorly differentiated tumors [14,15].

Aberrant expression of specific miRNAs in GC has been previously reported and was correlated with the progression and prognosis of GC [16-18]. We previously found that miR-24, which can serve as a tumor suppressor through a target site polymorphism [19], was a significantly downregulated miRNA in GC cells and tissues compared with non-tumor tissues. In this study, we analyzed miR-24 expression levels in GC tissues compared with matched non-tumor tissues, and assessed correlations between miR-24 level and clinicopathologic parameters. We evaluated the influence of overexpression of miR-24 on the growth and apoptosis of SGC-7901 GC cells both *in vitro* and *in vivo*. We also determined the biological effect of downregulation of miR-24 expression on GC cells. Furthermore, we identified RegIV (regenerating islet-derived family, member 4) as one of the target genes of miR-24. We hypothesize that miR-24 can regulate the invasion and metastasis of GC cells by directly targeting the RegIV gene.

## Results

### Overexpression of miR-24 inhibits GC cell proliferation

**Figure 1:**
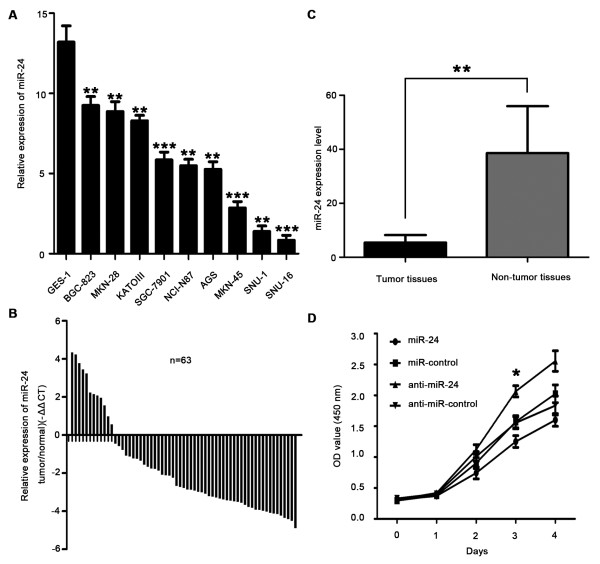
**miR-24 is significantly downregulated in GC and inhibited SGC-7901 cell growth. (A)** Relative expression of miR-24 in nine GC cell lines compared with GES-1 determined by qRT-PCR of three independent experiments (**P < 0.01, ***P < 0.001). **(B)** Relative expression of miR-24 in surgical specimens of 63 GC determined by qRT-PCR. All experiments were performed in triplicate. Data are shown as -ΔΔCT values, S.D. not shown. **(C)** The mean and standard deviation of miR-24 expression levels in surgical specimens of 63 GC tissues and matched non-tumor tissues are shown. Data are presented as 2^-ΔCT^ values (**P < 0.05). U6 snRNA was used for normalization. **(D)** miR-24 inhibited the growth of SGC-7901 cells as detected by WST, while anti-miR-24 increased cell growth (*P < 0.05). Data are shown as mean ± S.D. of three independent experiments.

To explore the expression of miR-24 in GC, quantitative real-time RT-PCR (qRT-PCR) was performed. Expression of miR-24 was generally downregulated in nine GC cell lines compared with the immortalized normal gastric mucosal epithelial cell GES-1 (Figure 1A). Expression of miR-24 was examined further with qRT-PCR in tumor tissues and matched non-tumor tissues from 63 GC patients (Figure 1B). The average expression level of miR-24 was significantly downregulated in tumor tissues compared to matched non-tumor tissues (Figure 1C). Together, these results provided strong evidence that miR-24 was significantly downregulated in GC.

We next examined the correlations between expression level of miR-24 and clinicopathologic factors in human GC. The clinical and pathologic characteristics of 63 GC patients are provided in Table 1. Based on relative expression ratios of miR-24/U6 = 1, the cases were divided into two groups: the miR-24 high-expression group (n = 29) and the miR-24 low-expression group (n = 34). The miR-24 low-expression group exhibited significantly lower tumor differentiation (P = 0.021). However, the miR-24 expression level did not show any relationship with age, gender, tumor site, the depth of local invasion, lymph node metastasis, or TNM stage.

**Table 1:** Relationship between miR-24 expression level and clinicopathologic variables in 63 GC patients

**Clinicopathologic parameters**	**miR-24 expression**		**P value**
	**High (n = 29)**	**Low (n = 34)**	
*Gender*			
Male	17	21	0.799
Female	12	13	
*Age (years)*			
<60	15	18	0.923
≥60	14	16	
*Tumor location*			
Distal third	8	8	0.310
Middle third	6	13	
Proximal third	15	13	
*WHO classification*			
Adenocarcinoma	25	28	0.155
Signet-ring cell carcinoma	1	5	
Mucinous adenocarcinoma	3	1	
*Differentiation*			
Well, moderately	15	8	0.021
Poorly	14	26	
*Borrmann classification*			
I/II	11	12	0.828
III/IV	18	22	
*Local invasion*			
T1/T2	1	7	0.098
T3/T4	28	27	
*Lymph node metastasis*			
No	6	4	0.535
Yes	23	30	
*Distant metastasis*			
No	28	31	0.724
Yes	1	3	
*TNM stage*			
I/II	7	8	0.955
III/IV	22	26	

To investigate the biological function of miR-24 in development and progression of GC, we transfected SGC-7901 cells with miR-24 mimics (SGC-7901/miR-24) or miR-24 inhibitor (SGC-7901/anti-miR-24). As shown in Additional file 1: Figure S1A and S1B, we selected 100 nM as suitable concentration in subsequent experiments. Ectopic expression of miR-24 in SGC-7901 cells was confirmed by qRT-PCR. Next, we examined cell proliferation by WST assay. Overexpression of miR-24 inhibited the growth rate of SGC-7901 cells compared with miR-control transfected cells (P < 0.05), whereas anti-miR-24 increased cell growth activities (P < 0.05, Figure 1D).

### Overexpression of miR-24 inhibits the migration and invasion of GC cells

**Figure 2:**
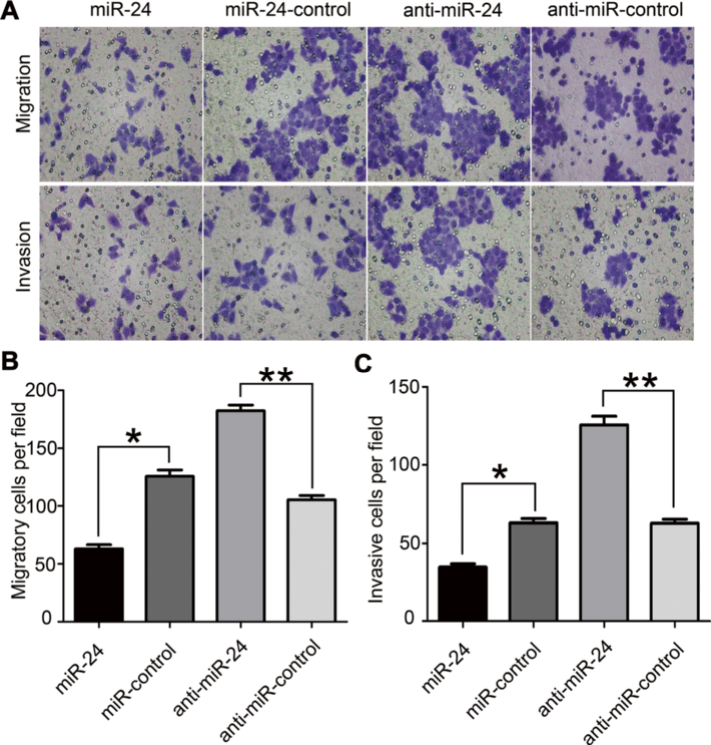
**miR-24 inhibited migration and invasion of SGC-7901 cells. (A)** Representative fields of SGC-7901 cells in the transwell assays. **(B, C)** Representative fields of SGC-7901 cells in the migration and invasion assays, respectively. Average number of migration and invasion cells per field from three independent experiments (*P < 0.05, **P < 0.01).

Next we evaluated the effect of miR-24 on cell migration and invasion in GC. Cell migration and invasion assays showed that overexpression of miR-24 suppressed cell migration (SGC-7901/miR-24 group, 63.0 ± 3.6 cells per field; control group, 126.3 ± 7.0 cells per field; P < 0.05) and invasion (SGC-7901/miR-24 group, 34.7 ± 2.1 cells per field; control group, 63.7 ± 3.5 cells per field; P < 0.05) (Figure 2A,B). In contrast, knockdown of miR-24 significantly increased cell migration (SGC-7901/anti-miR-24 group, 182.3 ± 5.0 cells per field; control group, 105.3 ± 3.8 cells per field; P < 0.01) and invasion (SGC-7901/anti-miR-24 group, 124.3 ± 3.8 cells per field; control group, 62.7 ± 2.5 per field; P < 0.01) (Figure 2A,C).

### Overexpression of miR-24 promotes the apoptosis of GC cells and induces cell cycle arrest in G0/G1 phase

**Figure 3:**
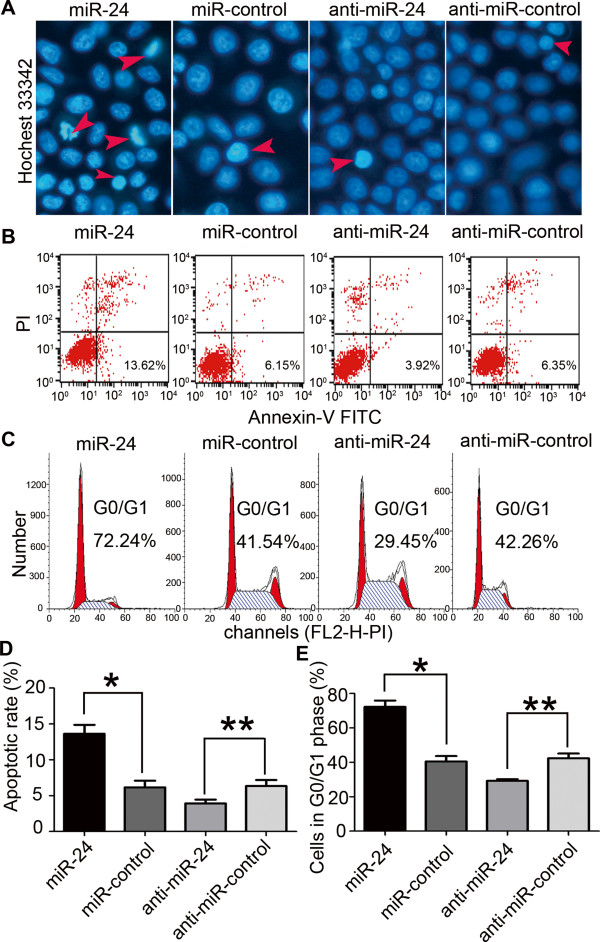
**miR-24 induced cell apoptosis and G0/G1 cell cycle arrest. (A)** Representative histograms depicting nuclear morphology of SGC-7901 cells transiently transfected with 100 nM miR-24 mimics or inhibitor and their respective controls (Hoechst33342 staining, original magnification 400×). **(B)** Representative histograms depicting apoptosis of SGC-7901 cells transiently transfected with 100 nM miR-24 mimics or inhibitor and their respective controls. **(C)** Representative histograms depicting cell cycle of SGC-7901 cells transiently transfected with 100 nM miR-24 mimics or inhibitor and their respective controls. **(D, E)** The percentage of apoptotic and G0/G1 phase cells of three independent experiments, mean ± S.D. (*P < 0.05, **P < 0.01).

Given that observed cellular growth may be affected by the rates of apoptosis and cell cycle progression, we examined the effects of miR-24 on apoptosis and cell cycle *in vivo* by flow cytometry. We found that approximately 12–15% of SGC-7901/miR-24 cells exhibited morphologic features typical of apoptosis, including condensed chromatin and nuclear fragmentation by Hoechst33342 staining for DNA content. In contrast, after accounting for the rare spontaneous apoptosis in SGC-7901 cells, the SGC-7901/anti-miR-24 group did not show any significant changes by Hoechst33342 staining (Figure 3A). Flow cytometry showed that the apoptotic rate was significantly increased in SGC-7901/miR-24 cells compared with control cells (13.62% ± 1.25% vs. 6.15% ± 0.95%, respectively; P < 0.01), and significantly decreased in SGC-7901/anti-miR-24 compared with controls (3.92% ± 0.52% vs. 6.35% ± 0.83%, respectively; P < 0.05) (Figure 3B,D).To further elucidate the mechanism of miR-24-mediated growth inhibition of GC cells, cell cycle analysis was performed (Figure 3C,E). Upon upregulation of miR-24, the percentage of cells in G0/G1 phase increased from 40.51% ± 3.15% in controls to 72.24% ± 3.65% (P < 0.01), while knockdown miR-24 reduced the percentage of cells in G0/G1 phase from 42.35% ± 2.78% in controls to 30.25% ± 1.25% (P < 0.05).

### RegIV is a target gene of miR-24

**Figure 4:**
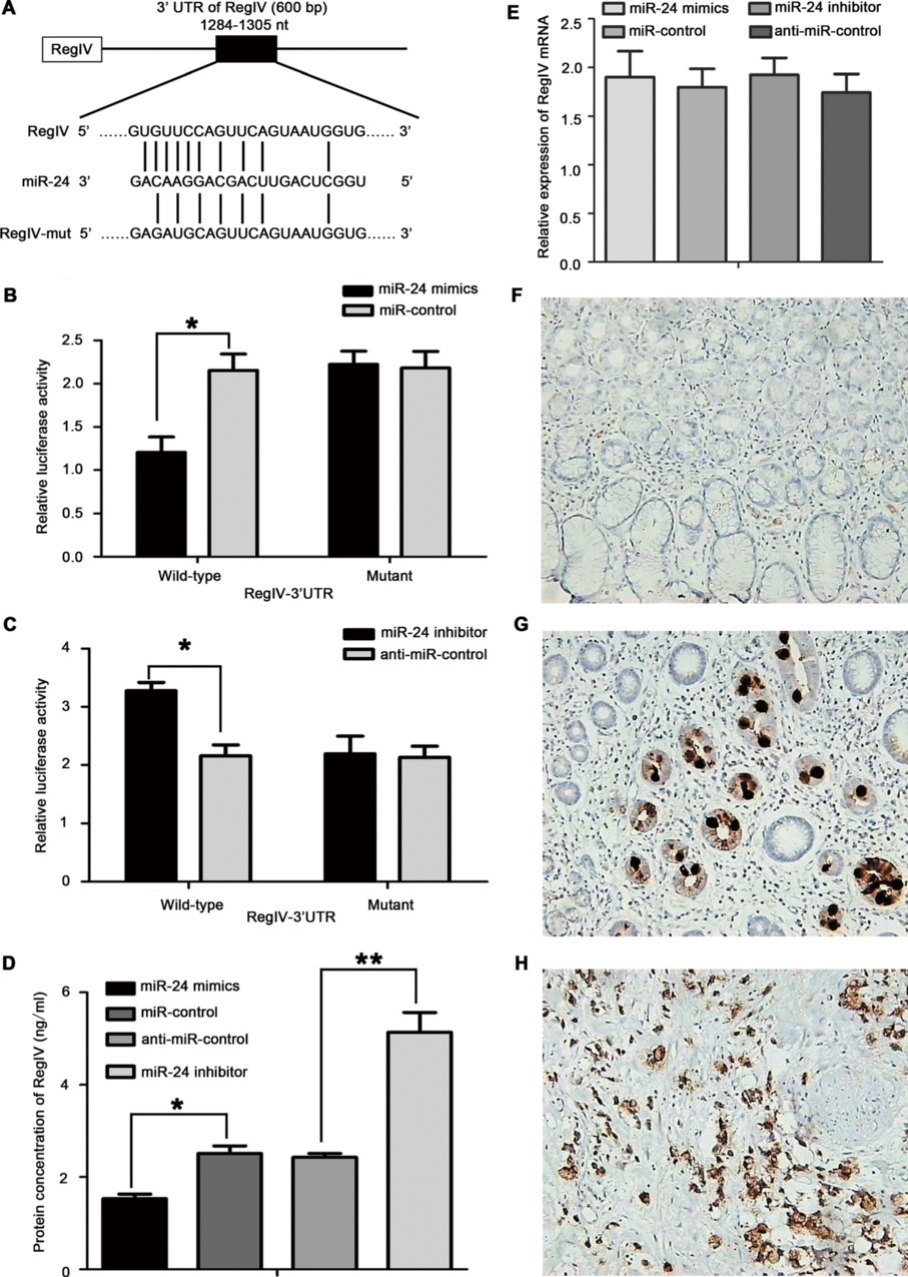
**miR-24 targeted the 3′UTR of RegIV gene and immunostaining of RegIV in gastric tissues. (A)** Schematic graph of the putative binding sites of miR-24 in the RegIV 3′UTR. RegIV-mut indicates the RegIV-3′UTR with mutation in miR-24-binding sites. **(B)** miR-24 mimics downregulated activity of a luciferase reporter containing wild-type RegIV 3′UTR (*P < 0.05), but not the reporter with mutant RegIV 3′UTR. **(C)** Anti-miR-24 increased the luciferase activity of wild-type Luc-RegIV (*P < 0.05), but had no effect on the mutant. **(D)** Forty-eight hours after miR-24 mimic and control transfection of SGC-7901 cells, the protein level of RegIV was significantly decreased compared with the miR-control as determined by ELISA. Anti-miR-24 increased the expression of RegIV compared with the anti-miR-control (*P < 0.05, **P < 0.01). **(E)** Twenty-four hours after miR-24 mimic or inhibitor and the respective control transfection in SGC-7901 cells, there was no difference in the mRNA level of RegIV compared with the miR-control as determined by qRT-PCR (P > 0.05). Data are shown as mean ± S.D. of three independent experiments. **(F)** No expression of RegIV was detected in normal gastric mucosa. **(G)** RegIV was expressed in intestinal metaplasia of stomach. **(H)** Strong expression of RegIV was observed in gastric signet-ring cell carcinoma.

To more closely examine the mechanisms of miR-24 in GC, we searched for candidate target genes by bioinformatics. TargetScan, miRBase Tatget and StarBase were applied to search for potential targets of miR-24. Among the predicted targets, RegIV was identified as one of the target genes of miR-24, and we identified one potential miR-24 binding site within its 3′UTR (Figure 4A). Next, we examined the expression of RegIV in nine GC cells and GES-1. We found that RegIV was overexpressed in nine GC cells compared with GES-1, and exhibited an inverse expression pattern compared with miR-24 (Additional file 2: Figure S2A and S2B). To investigate whether the 3′UTR of RegIV mRNA was a functional target of miR-24, luciferase reporter gene assays were performed. We first evaluated the activity of miR-24 (or miR-control) co-transfected into SGC-7901 cells with Luc-RegIV plasmid or the Luc-RegIV-mut plasmid (in which the putative miR-24 binding site was mutated), along with the pRL-TK plasmid containing the Renilla luciferase gene as an internal control (Figure 4B). Cells co-transfected with miR-24 demonstrated a significant decrease of luciferase activity compared with the miR-control group (P < 0.05). However, miR-24 co-transfected with the Luc-RegIV-mut plasmid showed no significant difference in reporter activity compared with cells co-transfected with miR-control. Likewise, anti-miR-24 increased the luciferase activity of wild-type Luc-RegIV, but had no effect on Luc-RegIV-mut plasmid (Figure 4C; P < 0.05). We also performed luciferase reporter gene assays in SNU-16 to minimize the influence of endogenous miR-24. At first, ectopic expression of miR-24 in SNU-16 cells was confirmed by qRT-PCR. Expression of miR-24 transfected with miR-24 mimics was about 50 times higher than that of miR-control group in SNU-16 (P < 0.01), while no statistical difference with the transfection of anti-miR-24 (Additional file 3: Figure S3A). Cells co-transfected with miR-24 demonstrated a significant decrease of luciferase activity compared with the miR-control group (P < 0.001), while no statistical difference with anti-miR-24 transfection (Additional file 3: Figure S3B and S3C). These results strongly indicated that the 3′UTR of RegIV contains direct binding sites for miR-24.

Next we examined miR-24 regulation of RegIV mRNA and protein levels in transfected SGC-7901 cells. ELISA analysis showed that RegIV protein levels were greatly suppressed in SGC-7901/miR-24 cells, whereas RegIV protein levels were upregulated in SGC-7901/anti-miR-24 cells (Figure 4D; *P < 0.05, **P < 0.01). qRT-PCR analysis indicated no difference in the level of RegIV mRNA in all transfected cell groups (Figure 4E; P > 0.05). Meanwhile, we detected the expression of c-MYC in GC cells as positive control transfected with miR-24 [20]. We found that miR-24 downregulated RegIV and c-MYC (Additional file 4: Figure S4A and S4B).

Previous studies demonstrated that RegIV was associated with proliferation and metastasis of GC. We next used immunohistochemical analysis to evaluate the expression of RegIV in GC. Our findings confirmed overexpression of RegIV in GC. The protein level of RegIV in normal gastric mucosa was lower than intestinal metaplasia of stomach and gastric signet-ring cell carcinoma (Figure 4F–H). To assess the clinical relevance of these findings, we examined the correlation between the expressions of RegIV and miR-24 in GC tissues. We found that RegIV and miR-24 exhibited an inverse expression pattern in GC tissues (Table 2). These results support the notion that downregulation of miR-24 resulted in increased protein levels of RegIV in GC.

**Table 2:** RegIV and miR-24 exhibit inverse expression pattern in GC

	**miR-24 expression**		**P value**
	**Low**	**High**	
Reg IV (IHC)			
Negative	9	16	0.038*
Positive	25	13	

*P <0.05 was considered statistically significant.

### Overexpression of miR-24 inhibits tumorigenicity in vivo

**Figure 5:**
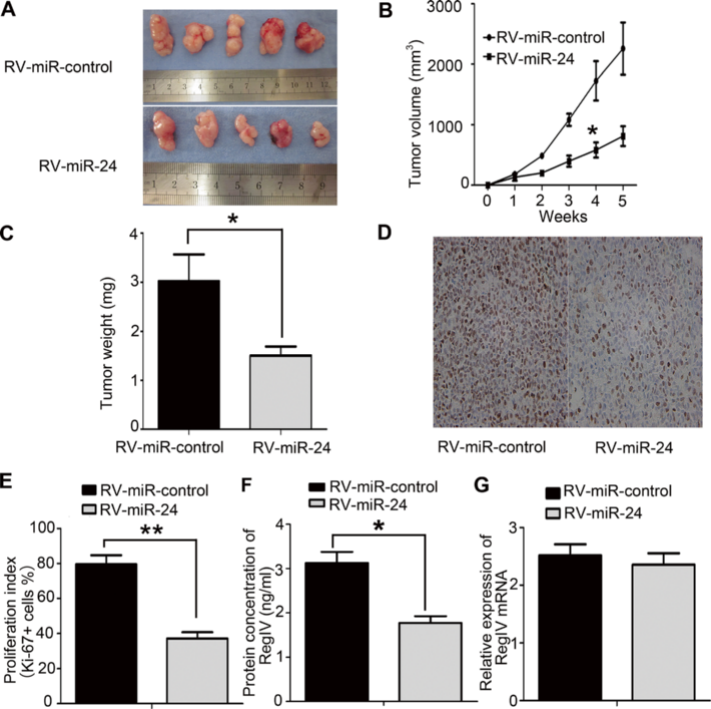
**miR-24 inhibited tumorigenicity and proliferation in vivo. (A)** Photograph of tumors derived from RV-miR-24 or RV-miR-control cells in nude mice. **(B)** Growth kinetics of tumors in nude mice. Tumor diameters were measured every 7 days. The volume of the nude mice indicated by bars, S.D. (*P < 0.05). **(C)** The average weight of tumors in nude mice. Data are shown as means ± S.D. (*P < 0.05). **(D)** Representative photographs of immunohistochemical analysis of Ki-67 antigen in tumors of nude mice (original magnification, 200×). **(E)** Representative proliferative index of tumors in RV-miR-24 and RV-miR-control nude mice. Data are shown as means ± S.D. (**P < 0.05). **(F)** Expression levels of RegIV in tumor tissues of nude mice. Data are shown as means ± S.D. (*P < 0.05). **(G)** Expression levels of RegIV mRNA in tumor tissues of nude mice (P > 0.05). Data are shown as mean ± S.D. of three independent experiments.

Given that miR-24 improved the proliferation of GC cells *in vitro*, we examined whether miR-24 could affect tumorigenicity *in vivo*. Retrovirus-mediated SGC-7901/miR-24 and SGC-7901/miR-control stable cell lines were obtained as described in the Methods section. SGC-7901/RV-miR-24 and SGC-7901/RV-miR-control cells were injected subcutaneously into four-week-old male nude mice, and tumor formation was monitored. Tumors grew slower in the SGC-7901/RV-miR-24 group than those in the SGC-7901/RV-miR-control group (Figure 5A). The average tumor volume in mice inoculated with SGC-7901/RV-miR-24 cells at day 28 was significantly smaller compared to mice inoculated with SGC-7901/RV-miR-control cells (397.45 ± 93.07 mm^3^ vs. 1083.56 ± 101.56 mm^3^, respectively) (Figure 5B and C; P < 0.05).

To assess whether tumor growth inhibition in SGC-7901/RV-miR-24 cells was partly due to the suppression of proliferation, immunohistochemical analyses of tumor tissues were performed. As shown with Ki-67 antigen staining, the decreased tumor growth in mice injected with SGC-7901/RV-miR-24 cells may be partially because of lower proliferation caused by the overexpression of miR-24. The percentage of Ki-67-antigen-positive cells was lower in the tumor derived from SGC-7901/RV-miR-24 cells than the tumor derived from SGC-7901/RV-miR-control cells (37.1% ± 3.6% vs. 79.5% ± 5.2%, respectively) (Figure 5D and E; P < 0.01). The expression level of RegIV as determined by ELISA was lower in the tumor derived from cells overexpressing miR-24 than the tumor derived from control cells (1.77 ± 0.15 ng/ml in SGC-7901/RV-miR-24 vs. 3.13 ± 0.25 ng/ml in SGC-7901/RV-miR-control) (Figure 5F; P < 0.05). However, there was no statistical difference in the relative expression of RegIV mRNA (Figure 5G). Therefore, the tumorigenicity of SGC-7901/RV-miR-24 cells were significantly reduced *in vivo*.

## Discussion

Accumulating evidence has supported important roles for miRNAs, which act either as tumor suppressors or oncogenes, in GC [21-23]. In addition, many miRNAs have been shown to exhibit therapeutic potential. Because of their function as master regulators of the genome and novel mechanism of action, miRNAs are considered a promising technology for therapeutic development [24]. miR-26a has antitumorigenic properties and potential therapeutic utility for liver cancer *in vitro* and *in vivo*, and was associated with rapid and sustained inhibition of cancer cell proliferation and highly specific induction of tumor cell death [25]. The specific mechanism of the roles of dysregulated miRNAs in gastric carcinogenesis remains elusive.

In this study, we demonstrated the inhibitory effects of miR-24 on tumor metastasis at the clinical, cellular and molecular level and in an experimental animal model. We provided detailed mechanistic experimental evidence for the role of miR-24 in GC by suppressing the expression of RegIV. Studies showed that miR-24 may act as oncogene in malignant effusions [26], oral carcinoma [27,28], prostate cancer [3] and lung cancer [29,30], but may act as a tumor suppressor in colon cancer [19] and retinoblastoma tumors [31]. Lal A et al. found that miR-24 inhibited cell proliferation and cell cycle progression by suppressing the expression of E2F2, MYC and other cell cycle regulatory genes by binding to “seedless” 3′UTR miRNA recognition elements [20]. Wu J et al. reported that transfection of miR-24 into GC cells reduced the expression of AE1 protein, which resulted in inhibiting cellular proliferation [32]. However, the complete underlying mechanisms for miR-24 in GC are still not clear.

Our report identified miR-24 as a candidate tumor suppressor in GC. We found downregulation of miR-24 expression both in GC tissues and cell lines. Overexpression of miR-24 in SGC-7901 GC cells significantly reduced proliferation and invasion both *in vitro* and *in vivo*, revealing the potential therapeutic effect of miR-24 in GC. The opposite result was obtained when the expression of miR-24 was inhibited by anti-miR-24. Together these results suggest that miR-24 may function as a tumor suppressor in human GC.

miRNAs bind to perfect or imperfect complementary “seed” sequences in target mRNAs, leading to cleavage of target mRNAs or inhibition of their translation [33]. In this study, we identified RegIV as a target gene of miR-24. miR-24 bound with incomplete complementarity to RegIV mRNA, resulting in direct translational inhibition of RegIV mRNA but with no effect on overall mRNA stability. As the different action modes of miRNA, miR-24 did not affect the mRNA level of RegIV but the translational inhibition. RegIV is a member of the human Reg gene family, which shares sequence similarity with the carbohydrate-binding domain of C-type lectins, and was first isolated and characterized in human inflammatory bowel disease [34]. RegIV is one type of secreted proteins, and plays a biological role depending on the extracellular secreted proteins, so that we took ELISA as a preferred detection method. Western blot was also performed to validate the protein level of RegIV, and we obtained the similar results. Previous studies have reported that the RegIV gene was frequently overexpressed in GC and is potentially involved in invasion, metastasis, and carcinogenesis of GC. RegIV expression was narrowly restricted in noncancerous tissues [35,36]. RegIV expression was markedly higher in patients with peritoneal metastasis compared to those without peritoneal metastasis. RegIV might accelerate peritoneal metastasis in GC and levels in lavage fluids could serve as a good marker for peritoneal metastasis [37-39]. RegIV may function as a serum biomarker for GC patients and inhibits 5-FU-induced apoptosis by induction of Bcl-2 and dihydropyrimidine dehydrogenase [40].

In the current study, we identified miR-24 as a potential upstream regulator of RegIV. First, overexpression of miR-24 decreased the activity of a luciferase reporter gene containing the 3′UTR of RegIV, while mutation of the “seed region” sites in the 3′UTR of RegIV abolished the regulatory effect of miR-24. The opposite result was obtained when we used anti-miR-24. Second, human GC tissues expressed significantly lower levels of miR-24 than non-tumor tissues, while GC tissues contain significantly higher levels of RegIV protein than non-tumor tissues. Third, overexpression of miR-24 downregulated RegIV at the protein level, while downregulation of miR-24 increased RegIV protein level. Fourth, overexpression of miR-24 was significantly related with proliferation and metastasis, indicating a functional overlap with RegIV. All these results indicate that RegIV might be a direct target gene of miR-24 in GC.

Carcinogenesis is a series of sequential events, including detachment, migration, local invasion, angiogenesis, intravasation, survival in the circulatory system, extravasation and regrowth in different organs [41,42]. Here we showed that miR-24 could regulate the carcinogenesis of GC through modulating proliferation, migration and local invasion. Furthermore, our evidence suggests the possibility for miR-24 as a therapeutic target in GC. Further studies are required to fully understand the detailed mechanisms of miR-24 in GC carcinogenesis and as a potential therapeutic approach.

## Methods

### Ethical statement

Written informed consent was collected from all participants, and study protocol was approved by the ethics committee of Ruijin Hospital, Shanghai Jiaotong University School of Medicine. All mouse experiments were approved by the Animal Care (Permit#20120810) and Use Committee and conducted in accordance with the official recommendations of the Care and Use Laboratory Animals of Ruijin Hospital, Shanghai Jiaotong University School of Medicine.

### Cell lines

The human GC cell lines SNU-1, SNU-16, NCI-N87, and KATO III were purchased from American Type Culture Collection (ATCC). The cell lines SGC-7901, MKN-28, BGC-823, MKN-45, and AGS were purchased from Shanghai Institutes for Biological Sciences, Chinese Academy of Sciences. The immortalized normal gastric mucosal epithelial cell line (GES-1) was a gift from Prof. Feng Bi (Huaxi Medical University, Chengdu, Sichuan Province, PR China). The embryonic kidney cell line 293 T was preserved in our laboratory and maintained in Dulbecco’s modified Eagle’s medium (DMEM) with 10% fetal bovine serum (FBS) at 37°C in a humidified atmosphere with 5% CO_2_. All GC cells were cultured in RPMI 1640 supplemented with 10% FBS in a humidified incubator with 5% CO_2_ at 37°C.

### Tissue samples

Primary cancer tissues and paired adjacent non-tumor tissues were collected from patients with GC underwent radical gastrectomy at the Department of Surgery, Ruijin Hospital, Shanghai Jiao Tong University School of Medicine. The adjacent normal tissues were obtained over 5 cm from the primary cancer. Tissue samples were immediately snap-frozen in liquid nitrogen and stored in a refrigerator at −80°C. Tissues for immunohistochemistry were fixed in 4% paraformaldehyde and paraffin-embedded. Clinicopathological data were reviewed, and TNM staging classification was based on criteria of American Joint Committee on Cancer (AJCC, 6th edition). All samples were verified by pathological examination.

### RNA isolation and qRT-PCR

Total RNA was isolated from tissue samples and cell lines using Trizol reagent (Invitrogen, Carlsbad, CA, USA) according to the manufacturer’s instructions. The expression levels of miRNAs were evaluated using the Hairpin-itTM miRNAs qPCR Quantitation Kit (GenePharma, Shanghai, PR China) following the manufacturer’s protocol. The U6 small nuclear RNA (RNU6B; GenePharma) was used for normalization. The relative expression ratio of miR-24 in each paired tumor and non-tumor tissue was calculated using the 2^-ΔΔCT^ method. The miR-24 expression level was defined as upregulated when the relative expression ratio was >1, and defined as downregulated when the relative expression ratio was <1. The expression level of RegIV and c-MYC mRNA was measured by qRT-PCR according to the instructions of the SYBR® Green PCR Master Mix (Applied Biosystems, Foster City, CA, USA). The primers for RegIV were 5′- CACGACCCACAGAAGAGGCAGC −3′ (forward) and 5′- GGCGCTTGTTGCATTCGTTGCT −3′ (reverse). Primers for c-MYC were 5′- TCTTCCCCTACCCTCTCAACGA −3′ (forward) and 5′- TTCCTCATCTTCTTGTTCCTCCTCA −3′ (reverse). Primers for GAPDH were 5′- GGACCTGACCTGCCGTCTAG −3′ (forward) and 5′- GTAGCCCAGGATGCCCTTGA −3′ (reverse) according to the human RegIV, c-MYC and GAPDH cDNA sequences in GenBank. The GAPDH mRNA level was used for normalization. PCRs of each sample were conducted in triplicate.

### Transient transfection

The hsa-miR-24 mimics (miR-24), negative control (miR-control), hsa-miR-24 inhibitor (anti-miR-24) and inhibitor negative control (anti-miR-control) oligonucleotides were purchased from GenePharma. Cells in logarithmic growth phase were trypsinized, counted, and seeded in 6-well plates to ensure 50% cell confluence on the next day for transfection. Transfection of cells with oligonucleotides was performed using Lipofectamine™ 2000 Reagent in line with the manufacturer’s instructions (Invitrogen) at a final concentration of 100 nM.

### ELISA

ELISA (Cusabio, Wuhan, China) analysis of RegIV was performed 48 h post-transfection with miR-24 oligonucleotides. The culture supernatants were collected, and the secreted RegIV level was quantified by ELISA according to the manufacturer’s protocol. The expression of RegIV in tumor tissues was detected by ELISA.

### Cell proliferation assay

SGC-7901 cells proliferation was measured by the colorimetric water-soluble tetrazolium salt (WST) assay with a Cell Counting Kit-8 according to the manufacturer’s instructions (Dojindo, Kumamoto, Japan). The cells were seeded in 96-well plates (2 × 10^3^ cells/well) 24 h post-transfection, and cell proliferation was examined every 24 h for 5 days. The number of viable cells was determined by measurement of the optical density difference between 450 nm and 600 nm using a Safire^2^ microplate reader (Tecan, Switzerland). All experiments were performed in triplicate.

### Migration and invasion assay

Migration assays were performed using the 6.5 mm Transwell® with 8.0 μm Pore Polycarbonate Membrane Insert (Corning, New York, USA) according to the manufacturer’s instructions. At 24 h post-transfection with miR-24 mimics, inhibitors and controls (100 nM), SGC-7901 cells were incubated in serum-free medium for 24 h, and then 3 × 10^4^ cells in 200 μl serum-free medium were added to the upper chamber. A volume of 500 μl of 10% FBS-containing medium was then added to the lower chamber as a chemoattractant. Cells were incubated for another 36 h at 37°C, and then non-migrating cells on the upper surface of the membrane were gently scraped off with cotton swabs. Cells that migrated to the bottom of the membrane were stained with the cell stain provided in the assay kit for 20 min and visualized under a microscope. To minimize the bias, at least three randomly selected fields with 200× magnification were counted, and the average number was taken.

For the invasion assay, 6.5 mm Transwell® with 8.0 μm Pore Polycarbonate Membrane Insert (Corning) was used according to the manufacturer’s instructions and matrigel (BD Biosciences, USA) was used to simulate the extracellular matrix in vivo. Briefly, 3 × 10^4^ SGC-7901 cells in 200 μl serum-free medium were added to the upper chamber pre-coated with matrigel gel. Then, 500 μl of 10% FBS-containing medium was added to the lower chamber as a chemo-attractant. Cells were incubated for 48 h at 37°C, and then non-invading cells were removed with cotton swabs. Invasive cells were fixed, stained with 0.04% crystal violet and counted as described above.

### Apoptosis and cell cycle analysis

At 48 h post-transfection, cells were collected by trypsinization and washed with PBS. For apoptosis analysis, cells were resuspended in Binding Buffer (Annexin V-FITC Apoptosis Detection Kit I, BD Pharmingen, USA) at a concentration of 1 × 10^6^ cells/ml. Next, 2.5 μl of FITC AnnexinV and 5 μl PI (BD Pharmingen) were added to 100 μl of the cell suspension. After incubation for 15 min in the dark at room temperature, 400 μ1 binding buffer was added. Apoptosis was analyzed by flow cytometry (FACSCalibur, Becton Dickinson) using Cell Quest software (Becton Dickinson). Annexin V-FITC-positive and PI-negative cells were detected as undergoing apoptosis.

For cell cycle analysis, the cells were fixed with 70% ethanol and stored at 4°C overnight. The following day, the fixed cells were washed with PBS, treated with 2 μl of RNase A (50 μg/ml), and stained with 20 μl of Propidium Iodide (50 μg/ml) for 30 min in the dark at 37°C, with rocking every 5 min. The stained cells were analyzed by flow cytometry (FACSCalibur). At least 10000 cells in each sample were analyzed to obtain a measurable signal.

For examining nuclear morphology associated with apoptosis, cells were washed in PBS and incubated with a DNA dye, Hoechst33342 (Beyotime, Nantong, PR China), according to the protocol of the kit. The staining was visualized under a fluorescent microscope that was excited at a wavelength of 350 nm and measured at 460 nm.

### Construction of the RegIV 3′UTR reporter gene system

A 600 bp fragment of the wild-type (WT) RegIV-3′UTR or mutant RegIV-3′UTR (mut) containing the putative miR-24 binding site was synthesized by RT-PCR. The wild-type RegIV-3′UTR was synthesized using the following sequences: 5′- ggactagtagcaagaatcaagattctgctaact −3′ (forward) and 5′- cgacgcgttgggtgtatttcttggtcttatttc −3′ (reverse). The mutant RegIV-3′UTR was designed to mutate three intermittent nucleotides complementary to the miR-24 seed region. The mutant sequences were as follows: 5′- gaggttgctgagatgcagttcagtaatggtgaatgtggaa −3′ (forward) and 5′- ttccacattcaccattactgaactggaacacagcaacctc −3′ (reverse). After digestion with SpeI and MluI, the wild-type and mutant RegIV-3′UTR fragments were cloned into the SpeI and MluI sites of the pMIR-Report luciferase vector (Applied Biosystems) and named pMIR/RegIV and pMIR/RegIV/mut, respectively. Proper insertion was confirmed by sequencing. SGC-7901 cells were seeded in 24-well plates. At 24 h later, cells were transfected with 200 ng of either pMIR/RegIV or pMIR/RegIV/mut, together with 2 ng of the pRL-TK vector (Promega, Madison, WI, USA) containing Renilla luciferase and 60 pmol of the miR-24 mimic, inhibitor or control. Transfection was performed using Lipofectamine™ 2000 (Invitrogen) and Opti-MEM I reduced serum medium. Cells were harvested 48 h post-transfection. Firefly and Renilla luciferase activities were measured by using a dual-luciferase reporter assay (Promega) according to the manufacturer’s protocol. Firefly luciferase activity was normalized to Renilla luciferase activity.

### Western blot analysis

Cells were lysed in RIPA buffer in the presence of Protease Inhibitor Cocktail (Pierce, Rockford, USA). Protein was quantified by a BCA Protein Assay Kit (Pierce, Rockford, USA). Protein (60 ug) was separated by 10% sodium dodecyl sulfate polyacrylamide gel electrophoresis, and transferred onto polyvinylidene fluoride membranes. The membranes were blocked with 5% non-fat milk in Tris-buffered saline and then incubated with primary antibodies at 4°C overnight. The primary antibodies used were anti-RegIV (1:1000; Abcam, USA), anti-c-MYC (1:500; Santa Cruz Biotechnology, Inc., USA) and anti-β-actin (1:5000; Abcam, USA). Membranes were then washed three times in TBST solution for 10 min each time, and then incubated with secondary antibodies. Signals were detected by an enhanced chemiluminescence detection system (Amersham Bioscience, Piscataway, NJ, USA) as the manufacturer’s protocol.

### Immunohistochemistry

Paraffin sections, 4 μm in thickness, were baked for 1 h at 68°C. After deparaffinization and rehydration, antigen retrieval was performed by boiling in 10 mmol/l of citrate buffer (pH 6.0) for 10 min. After inhibition of endogenous peroxidase activity for 30 min with methanol containing 0.3% H_2_O_2_, the sections were blocked with 2% bovine serum albumin in PBS for 30 min and incubated with mouse anti-human RegIV monoclonal antibody (Abcam, Hong Kong; dilution 1:150). The immune complex was visualized by the Dako REAL™EnVision™ Detection System, Peroxidase/DAB, Rabbit/Mouse (Dako, Denmark), according to the manufacturer’s procedures. The nuclei were counterstained with hematoxylin.

### Retroviral transfection for stable cell lines

The genomic region that included the primary transcript of miR-24 was cloned into the EcoRI-XhoI site of the modified pMSCV-GW-RfA-PGK-EGFP retroviral vector. Negative control vectors had no insert. For each cultured 293 T plate (10 cm), a plasmid mixture containing 10 μg of miR-24 retroviral vector, 10 μg of gag/pol vector and 10 μg of VSVG vector was co-transfected with 90 μl FuGENE6 transfection reagent (Roche, Basel, Switzerland) added directly to 0.6 ml of serum-free medium. The plasmid/medium/FuGENE6 mix was added drop-wise to the 293 T plate. After 12 h, 15 ml viral collection medium was added to the transfected cells. We then harvested the virus twice a day for two days. Infections of SGC-7901 cells were performed in the presence of 8 μg/mL of polybrene in each well of a 6-well plate. SGC-7901 cells were spin infected at 1500 rpm for 0.5 h at room temperature and the virus-containing supernatant was removed after 2 h. Positive cells were selected by GFP expression by FACS and named as RV-miR-24 or RV-miR-control. Expression of miR-24 was confirmed by qRT-PCR.

### Tumor xenograft model

A total volume of 100 μl of cells (2 × 10^6^ cells) transfected with RV-miR-24 or RV-miR-control were inoculated subcutaneously into 4-week-old male nude mice (Institute of Zoology, Chinese Academy of Sciences, Shanghai, China). Mice were checked weekly, and tumor nodules were measured with a caliper. Tumor volume was evaluated using the following formula: volume = (width + length)/2 × width × length × 0.5236. Tumor growth curves and inhibiting rates were calculated. The two experimental groups were sacrificed after 5 weeks. All tumor grafts were excised, weighed, harvested, fixed, and embedded. The mouse-anti-human Ki-67 antigen monoclonal antibody (Dako, dilution 1:50) was used to determine nuclear expression of Ki-67 antigen with immunohistochemistry procedures and samples were observed using a Nikon microscope (Nikon, Japan). The proliferative index (PI) score was measured by the mean percentage of nuclei staining positive for Ki-67 antigen in 1000 cells.

### Statistical analysis

The relationship between miR-24 expression level and clinicopathologic parameters was explored by the Pearson *X*^2^ test. The differences between groups were analyzed using Student *t* test when there were only two groups, or assessed by one-way ANOVA when there were more than two groups. All statistical analyses were performed using the SPSS 16.0 software. A two-tailed value of *P* < 0.05 was considered statistically significant.

## Competing interests

The authors declare that they have no competing interests.

## Authors’ contributions

YD, LH, MY, QY, BL, YY, CL, LS, MX and ZZ conceived and designed the experiments. YD, JL, BY, BL and LH performed the experiments. YD, LH and BL analyzed the data. YD, QY and BL wrote the manuscript. All authors read and approved the final manuscript.

## Supplementary Material

Additional file 1: Figure S1 To validate the transfection efficiency of miR-24 mimics and inhibitor in SGC-7901. (A) 1nM, 50 nM, 100 nM miR-24 mimics and control transfected SGC-7901 (*P < 0.05, ***P < 0.001). (B) 1 nM, 50 nM, 100nM miR-24 inhibitor and control transfected SGC-7901. U6 snRNA was used for normalization. Data are shown as mean ± S.D. of three independent experiments (**P < 0.01). Click here for file

Additional file 2: Figure S2 Expression of RegIV in mRNA and protein levels. (A) Elevated expression of RegIV mRNA in nine GC cell lines compared with GES-1 determined by qRT-PCR. Data are shown as -ΔΔCt values (*P < 0.05, **P < 0.01, ***P < 0.001). GAPDH was used for normalization. (B) Elevated expression of RegIV protein in nine GC cell lines compared to GES-1 determined by ELISA. Data are shown as mean ± S.D. of three independent experiments (*P < 0.05, **P < 0.01, ***P < 0.001). Click here for file

Additional file 3: Figure S3 miR-24 targeted RegIV in SNU-16. (A) miR-24 expression in SNU-16 cells was effectively elevated by transient transfection of miR-24 (miR-24 mimics), while no statistical difference by anti-miR-24 (miR-24 inhibitor) as detected by qRT-PCR (**P < 0.01). (B) miR-24 mimics downregulated activity of a luciferase reporter containing wild-type RegIV 3′UTR (***P < 0.001), but not the reporter with mutant RegIV 3′UTR. (C) Anti-miR-24 had no statistical difference on luciferase activity of wild-type or mutant Luc-RegIV. Data are shown as mean ± S.D. of three independent experiments. Click here for file

Additional file 4: Figure S4 miR-24 downregulated c-MYC expression in GC cell. (A) miR-24 downregulated c-MYC mRNA level in SGC-7901 and SNU-16. Data are shown as mean ± S.D. of three independent experiments (**P < 0.01). (B) miR-24 downregulated c-MYC and RegIV protein levels in SGC-7901 and SNU-16. Click here for file
