# Supplementary figures and images for: Tumor suppressor miR-24 restrains gastric cancer progression by downregulating RegIV
Source: Mol Cancer. 2014 May 28;13:127. doi: 10.1186/1476-4598-13-127 (PMC4041902; doi:10.1186/1476-4598-13-127)

**A**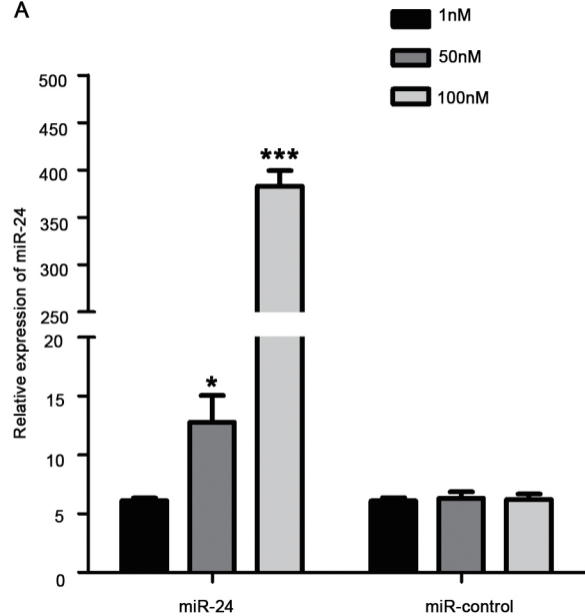**B**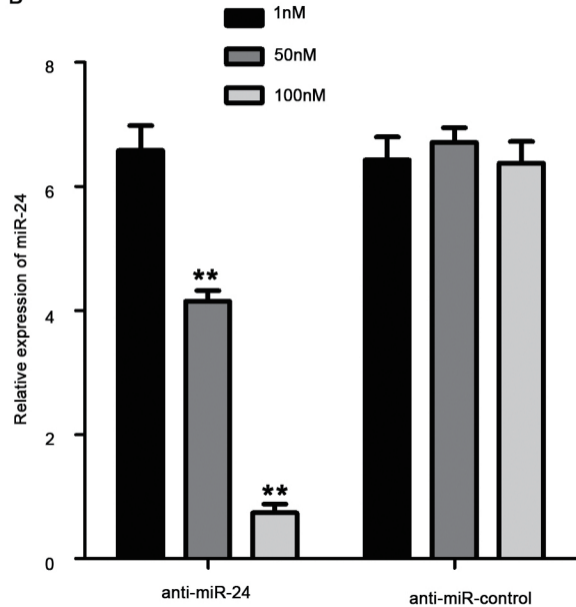

Supplement: Additional file 1: Figure S1 — To validate the transfection efficiency of miR-24 mimics and inhibitor in SGC-7901. (A) 1nM, 50 nM, 100 nM miR-24 mimics and control transfected SGC-7901 (*P < 0.05, ***P < 0.001). (B) 1 nM, 50 nM, 100nM miR-24 inhibitor and control transfected SGC-7901. U6 snRNA was used for normalization. Data are shown as mean ± S.D. of three independent experiments (**P < 0.01). [file 1476-4598-13-127-S1.pdf]

**A**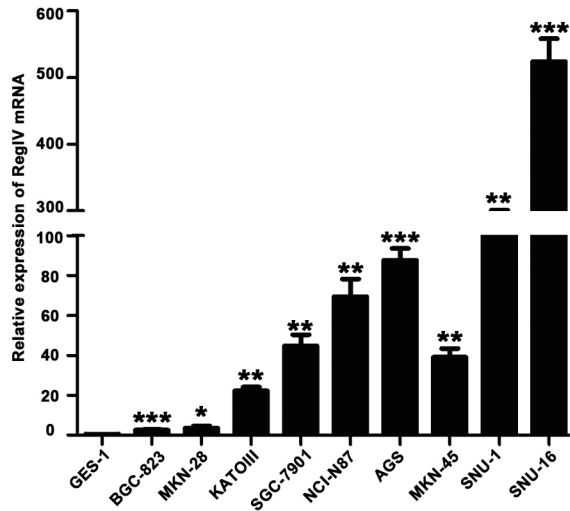**B**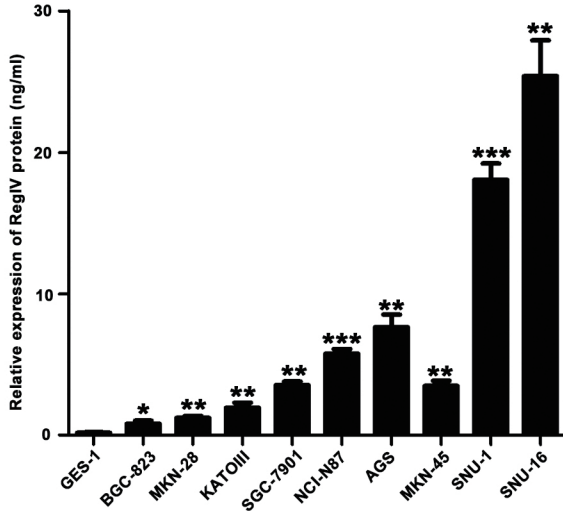

Supplement: Additional file 2: Figure S2 — Expression of RegIV in mRNA and protein levels. (A) Elevated expression of RegIV mRNA in nine GC cell lines compared with GES-1 determined by qRT-PCR. Data are shown as -ΔΔCt values (*P < 0.05, **P < 0.01, ***P < 0.001). GAPDH was used for normalization. (B) Elevated expression of RegIV protein in nine GC cell lines compared to GES-1 determined by ELISA. Data are shown as mean ± S.D. of three independent experiments (*P < 0.05, **P < 0.01, ***P < 0.001). [file 1476-4598-13-127-S2.pdf]

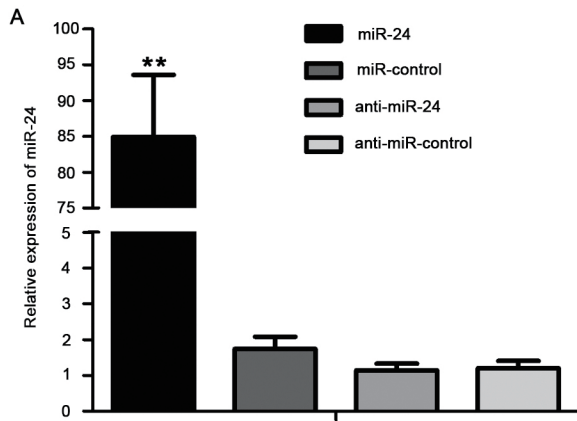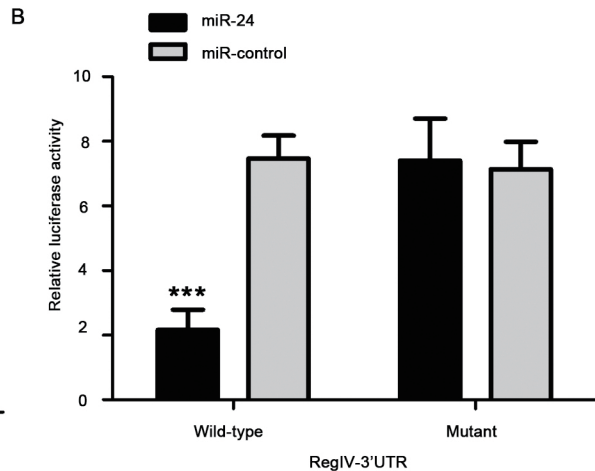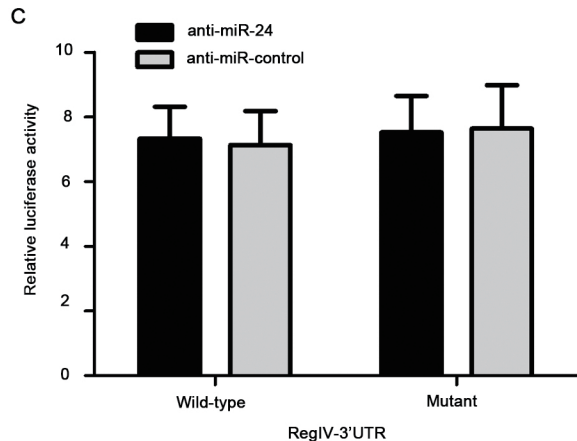

Supplement: Additional file 3: Figure S3 — miR-24 targeted RegIV in SNU-16. (A) miR-24 expression in SNU-16 cells was effectively elevated by transient transfection of miR-24 (miR-24 mimics), while no statistical difference by anti-miR-24 (miR-24 inhibitor) as detected by qRT-PCR (**P < 0.01). (B) miR-24 mimics downregulated activity of a luciferase reporter containing wild-type RegIV 3′UTR (***P < 0.001), but not the reporter with mutant RegIV 3′UTR. (C) Anti-miR-24 had no statistical difference on luciferase activity of wild-type or mutant Luc-RegIV. Data are shown as mean ± S.D. of three independent experiments. [file 1476-4598-13-127-S3.pdf]

**A**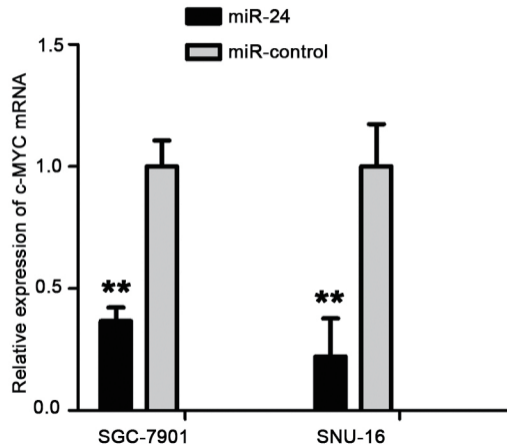**B**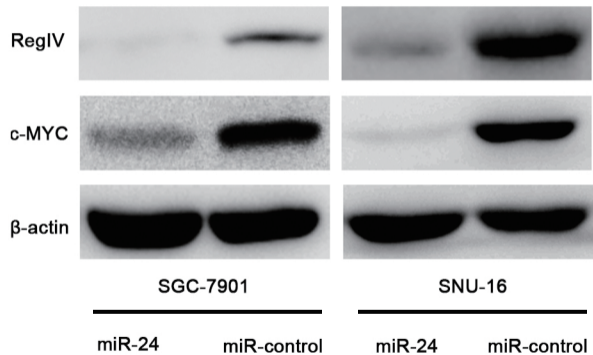

Supplement: Additional file 4: Figure S4 — miR-24 downregulated c-MYC expression in GC cell. (A) miR-24 downregulated c-MYC mRNA level in SGC-7901 and SNU-16. Data are shown as mean ± S.D. of three independent experiments (**P < 0.01). (B) miR-24 downregulated c-MYC and RegIV protein levels in SGC-7901 and SNU-16. [file 1476-4598-13-127-S4.pdf]
